# Impact of fibronectin type III domain-containing family in the changes in metabolic and hormonal profiles during peripartum period in dairy cows

**DOI:** 10.3389/fvets.2022.960778

**Published:** 2022-07-27

**Authors:** Mathilde Daudon, Christelle Ramé, Anthony Estienne, Christopher Price, Joëlle Dupont

**Affiliations:** ^1^CNRS, IFCE, INRAE, Université de Tours, PRC, Breeding system and Animal Physiology Department, Nouzilly, France; ^2^Centre de Recherche en Reproduction et Fertilité, Faculté de Médecine Vétérinaire, Université de Montréal, St-Hyacinthe, QC, Canada

**Keywords:** fibronectin type III domain-containing proteins, irisin, negative energy balance, dairy cows, adipokine, myokine

## Abstract

The peripartum period in dairy cows is frequently associated with excessive lipolysis due to Negative Energy Balance (NEB). These metabolic disorders are the cause of various pathologies. Some metabolites such as β-hydroxybutyrate (BHBA) and Non-Esterified Fatty Acids (NEFA) are known to be biomarkers of NEB in dairy cows. The involvement of adipokines, including adiponectin and leptin, during fat mobilization in the peripartum period is well described, but little is known about the impact of myokines at this time. Fibronectin type III domain-containing proteins (FNDC) are myokines and adipokines recently discovered to play a role in metabolic dysfunctions. This study aimed to evaluate some FNDCs (FNDC5, 4, 3A and B) as potential plasma and adipose tissue indicators of NEB in cattle. We measured plasma FNDC concentrations and adipose tissue FNDC gene expression during the peripartum period, 4 weeks before the estimated calving day (4WAP), one (1WPP) and 16 (16WPP) weeks postpartum in two groups of dairy cows with low NEB (LNEB, *n* = 8) and high NEB (HNEB, *n* = 13) at 1WPP. Using specific bovine ELISAs, only plasma FNDC5 concentrations varied during the peripartum period in both LNEB and HNEB animals; concentrations were higher at 1WPP as compared to 4WAP and 16 WPP. FNDC5 plasma concentrations was negatively correlated with dry matter intake, live body weight, variation of empty body weight and glucose concentrations, and positively correlated with plasma non-esterified fatty acids and BHBA concentrations. Subcutaneous adipose tissue contained abundant FNDC5 mRNA and protein, as measured by RT-qPCR and immunoblotting, respectively. We also observed that *FNDC5* mRNA abundance in subcutaneous adipose tissue was higher at 1 WPP as compared to 4WAP and 16WPP in HNEB cows and higher at 1 WPP as compared to 4 WAP in LNEB cows, and was higher in HNEB than in LNEB animals during early lactation. Finally, we showed that recombinant human irisin (a fragmented product of FNDC5) increased the release of glycerol and abundance of mRNA encoding adipose triglyceride lipase and hormone-sensitive-lipase in bovine and human adipose tissue explants. In conclusion, FNDC5 is expressed in bovine adipose tissue and may be involved in lipid mobilization and regulation of NEB in cattle.

## Introduction

At the beginning of lactation, a massive change in energy balance occurs in dairy cows resulting in mobilization of adipose and muscle tissues (1). The period between 3 weeks before and 3 weeks after parturition is defined as the “transition period” (2) and it is a key period for the next breeding season (1). Many metabolic adaptations occur with the onset of lactation when the energy requirements for milk production exceed energy consumption and the animal enters a state of negative energy balance (NEB). Although NEB is a physiological adaptation, it has a deleterious impact on the postpartum recovery of ovarian activity and fertility (1) and lactation performance (3) if the animal cannot cope with the severity and magnitude of NEB. The average duration of postpartum NEB is ~45 days [with a standard deviation of 21 days; (4)]. NEB leads to a mobilization of adipose tissue and the release of non-esterified fatty acids (**NEFA**) into the blood (5), which are the first two physiological adaptations to appear in order to meet the energy requirements of lactation. The liver removes circulating NEFA in the blood by metabolizing them into ketone bodies (6). An excess of ketone bodies in the blood leads to ketosis that can affect milk production (7) and fertility (5), with reproductive disorders affecting both the morphology of the cumulus oocyte complex and the viability of the embryo (8), increased uterine/placental incompetence (9) and an increased incidence of metritis and mastitis (10). The major circulating ketone body in cows is β-hydroxybutyrate (**BHBA**) and increased blood concentrations of BHBA is a marker of ketosis (11). During the peripartum period, lipogenesis is decreased and lipolysis is increased in adipose tissue by various physiological processes. For example, plasma insulin and glucose concentrations are decreased and concentrations of catecholamines, growth hormone, and glucocorticoids are increased (12). Furthermore, the profiles of many adipokines such as leptin, adiponectin (13–16), resistin (17, 18) and other biomarkers of lipid metabolism and inflammation (19) have been investigated in serum and/or adipose tissue of dairy cows during the periparturient period. Indeed, it has been shown that plasma leptin decreased before parturition and during period of undernutrition (20) and in opposite plasma resistin increased after parturition (13). Plasma adiponectin decreased before calving and increased after parturition (13). Moreover, glycerol release and adipose triglyceride lipase (ATGL) and hormone sensitive lipase (HSL) are involved in basal lipolysis (21). However, the transition period is also associated with the mobilization of muscle tissue (22–24), although this is secondary to fat mobilization (25, 26). Muscles secrete myokines, but whether myokine concentrations change during the peripartum period is unknown.

Recently, a new group of afipokines/myokines has been described, the fibronectin type III domain containing (FNDC), A family of membrane protein. Their various functions include tissue development and cell adhesion, migration and proliferation (27). This family contains FNDC3A, FNDC3B, FNDC4 and FNDC5 among others (28–31). FNDC5 is the most studied because it is the precursor of irisin, discovered in 2012 as a myokine involved in the regulation of thermogenesis in white adipose tissue in response to cold and exercise in mice and humans (32) by upregulating the Peroxisome Proliferator Activated Receptor γ (PPARγ) and Uncoupling Protein 1 (UCP1) (31, 33). Thus, irisin could be considered as a browning inducing endocrine hormone (32, 34, 35), presumably acting *via* integrin subunit beta 1 (ITGB1) and integrin subunit alpha V (ITGAV) as receptors (36) and the mitogen-activated protein kinase 3/1 (MAPK3/1) signaling pathway. It has also been suggested that full-length transmembrane FNDC5 could act as a receptor in the HEK-293 cell line (37). FNDC3B, also known as Factor for Adipocyte Differentiation 104 (Fad104), stimulates adipogenesis in mice (38) and its paralogue FNDC3A is expressed in Leydig cells and spermatids and is necessary for sperm maturation in mice (39). Similarly to FNDC5, FNDC4 is cleaved and releases a functional active peptide in the bloodstream that has recently been identified as an adipokine in humans (40). Abundance of *FNDC4* mRNA is elevated in adipose tissue of obese patients, and exogenous FNDC4 inhibited lipogenesis in human visceral adipocytes *in vitro* through the adhesion G-protein-coupled receptor F5 [ADGRF5, also known as G-protein-coupled receptor 116 (GPR116)] (40, 41) and upregulates UCP1 as well as FNDC5 (40).

As the four FNDCs described above are adipokines/myokines, we hypothesize that these FNDCs are linked to the NEB and the fat mobilization occurring during the transition period in the dairy cow. The objectives of the present study were to determine the tissue distribution of FNDC gene expression in cattle, and to determine adipose mRNA abundance and plasma concentrations of these FNDCs during the peripartum period and their correlations with zootechnical parameters, plasma metabolites and hormone profiles. Finally, we investigated the involvement of FNDC5/irisin in lipolysis of bovine adipose tissue by using explant cultures.

## Materials and methods

### Ethical issues

All experimental procedures were approved by an Ethics Committee (Comité d'Ethique en Expérimentation Animale Val de Loire, CEEA VdL, protocol reference number 2012-10-4) and were consistent with the guidelines provided by the French Council for Animal Care.

### Animals, experimental design and tissue collection

The study took place at the experimental unit UEPAO (Unité Expérimentale de Physiologie Animale de l'Orfrasière, Institut national de recherche pour l'agriculture, l'alimentation et l'environnement, Nouzilly, France).

**Figure 1:**
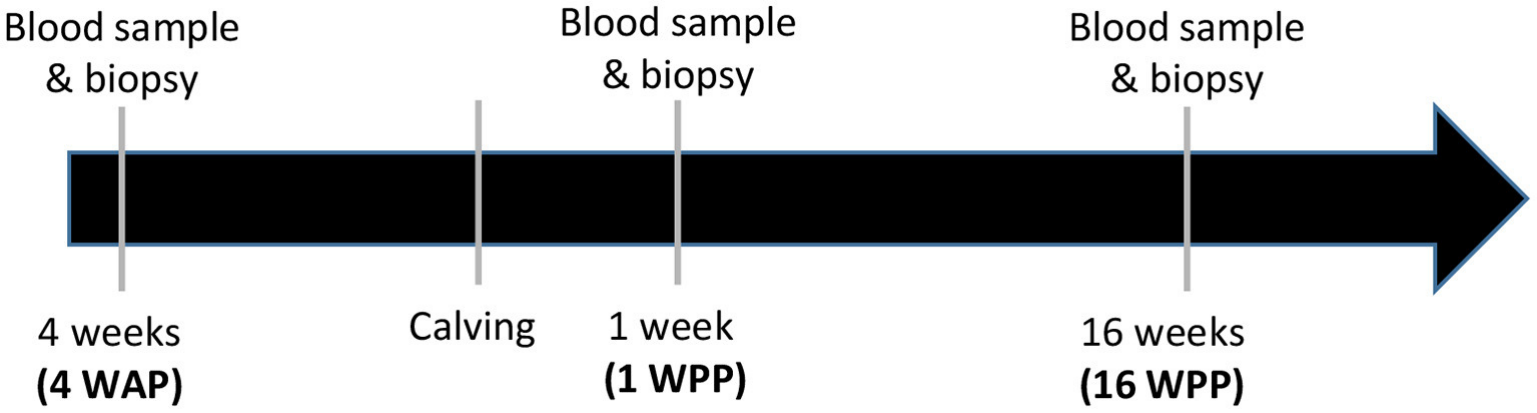
Description of the timing for biopsies and blood samples of Holstein dairy cows with different negative energy balance at 1 WPP (HNEB or LNEB).

From a herd of 39 Holstein heifers, two sub-groups of animals were selected with low negative energy balance (LNEB, *n* = 8) and high negative energy balance (HNEB, *n* = 13) according to their energy balance (EB) at 1 week postpartum [WPP, EB >-9 Mcal/day for LNEB and EB < -9 Mcal/ day for HNEB (42)] and to their plasma BHBA concentrations at 1 WPP [BHBA <1.2 mmol/L for LNEB and BHBA > 1.2 mmol/L for HNEB (11)]. Animals in the LNEB group were fed with a diet calculated to yield 35 kg of milk/cow/day and animals in the HNEB group were fed with a diet calculated to yield 25 kg of milk/cow/day (13). The diet composition for the LNEB group was: corn silage (61.2%), lucerne hay (10.1%); grass silage (0%), energy concentrate (17.2%), protein concentrate (10.7%), ca carbonate (0.5%) and minerals (0.3%) whereas those of HNEB was: corn silage (50.4%), lucerne hay (10.2%); grass silage (24%), energy concentrate (8.5%), protein concentrate (6%), ca carbonate (0.4%) and minerals (0.5%). Animals were reared in the same straw-bedded yard and the calving took place during the same period. The initial weight at 4 weeks before calving [4 weeks antepartum (4 WAP)] was 687.44 ± 18.91 kg for LNEB and 658.85 ± 18.34 kg for HNEB animals (Table 1). Blood samples and subcutaneous adipose tissue biopsies from cows were performed 4 WAP, 1 and 16 WPP (Figure 1). Cows were observed on their general condition in relation to feed intake, weariness, milk production and mammary infections every day; no clinical signs were apparent and no preventive medication was provided. Animals were routinely vaccinated against ovine catarrhal fever at 5 months of age with two injections 3–4 weeks apart and another 12 months later. During the dry period, cows were also vaccinated against neonatal diarrhea in calves (enrichment of colostrum). All the animals were artificially inseminated and propylene glycol was distributed at the beginning of lactation to avoid ketosis.

**Table 1:** Zootechnical parameters and plasma metabolites and hormonal concentrations in the high negative energy balance (HNEB, *n* = 13) and low negative energy balance (LNEB, *n* = 8) dairy cow groups^1^ at 4 WAP, 1 WPP and 16 WPP.

**Parameter**	**HNEB**			**LNEB**			***p*** **-Value**		
	**4 WAP**	**1 WPP**	**16 WPP**	**4 WAP**	**1 WPP**	**16 WPP**			
	**Mean** ±**SEM**	**Mean** ±**SEM**	**Mean** ±**SEM**	**Mean** ±**SEM**	**Mean** ±**SEM**	**Mean** ±**SEM**	**NEB level** **at 1 WPP**	**Time**	**NEB level** × **Time**
Energy balance (Mcal/day)	ND	−12.16^aA^ ± 0.53	−8.33^bA^ ± 1.84	ND	−5.60^aB^ ± 0.77	0.11^bB^ ± 1.43	<0.0001	0.0010	0.5832
Dry matter intake (kg/day)	ND	12.20^aA^ ± 1.4	15.51^bA^ ± 1.24	ND	13.090^aA^ ± 1.75	22.16^bB^ ± 1.69	0.0049	0.0002	0.0849
Live body weight (kg/day)	658.85^a^ ±18.4	564.35^b^ ± 17.74	525.75^b^ ± 14.63	687.44^a^ ± 18.91	602.76^b^ ± 19.94	569.27^b^ ± 15.83	0.0151	<0.0001	0.9155
Variation of empty body weight (kg/day)	0^a^	−14.39^b^ ± 1.07	−20.13^c^ ± 1.02	0^a^	−12.49^b^ ± 1.84	−17.4^a, b, c^ ± 2.15	0.1202	<0.0001	0.5099
Milk yield (kg/day)	ND	24.20 ^a^ ± 1.82	30.2 ^b^ ± 1.59	ND	21.60 ^a^ ± 2.13	33.5^b^ ± 2.30	0.8585	<0.0001	0.1383
Fat energy corrected milk (kg/day)	ND	4.6 ± 0.3^a^	3.9 ± 0.1^b^	ND	4.4 ± 0.3	4.1 ± 0.2	0.9259	0.0173	0.4071
Protein energy corrected milk (kg/day)	ND	2.7 ± 0.1	2.6 ± 0.05^A^	ND	2.9 ± 0.1	2.9 ± 0.04^B^	0.0094	0.4941	0.5162
Back fat thickness (cm)	0.41^a^ ± 0.04	0.34^a^ ± 0.03	0.17^b^ ± 0.01	0.46^a^ ± 0.07	0.44^a^ ± 0.04	0.21^b^ ± 0.01	0.0411	<0.0001	0.6893
Glucose (mmol/L)	6.90^a^ ± 0.45	4.48^b^ ± 0.34	6.35^a^ ± 0.48	5.67 ± 0.5	5.5 ± 0.42	5.28 ± 0.68	0.3038	0.0359	0.0359
NEFA (mmol/L)	0.33^a^ ± 0.05	1.54^b^ ± 0.11	0.51^a^ ± 0.14	0.25 ^a^ ± 0.06	1.43^b^ ± 0.42	0.96^a, b^ ± 0.24	0.5735	<0.0001	0.2519
BHBA (mmol/L)	0.42^aA^ ± 0.01	1.31^bA^ ± 0.03	0.41^aA^ ± 0.02	0.35^aA^ ± 0.01	1.16^bB^ ± 0.11	0.40^aA^ ± 0.02	0.0291	<0.0001	0.2549
Insulin (ng/ml)	0.2 ± 0.02	0.14 ± 0.04	0.11 ± 0.03	0.31 ± 0.12	0.21 ± 0.11	0.25 ± 0.08	0.0541	0.4086	0.8701
Leptin (ng/ml)	1.57^aA^ ± 0.04	0.74^bA^ ± 0.03	0.81^bA^ ± 0.01	1.66^aA^ ± 0.08	0.98^bB^ ± 0.06	1.22^bB^ ± 0.10	<0.0001	<0.0001	0.0160
Adiponectin (μg/ml)	5.04^aA^ ± 0.26	2.79^bA^ ± 0.18	4.68^aA^ ± 0.16	5.26^aA^ ± 0.19	2.59^bA^ ± 0.2	6.12^aB^ ± 0.37	0.0134	<0.0001	0.0025
Resistin (ng/ml)	41.82^aA^ ± 1.71	71.28^bA^ ± 1.35	31.82^cA^ ± 0.7	41.56^aA^ ± 2.39	63.78^bB^ ± 2.27	29.69^cA^ ± 0.56	0.0139	<0.0001	0.0701

^1^Results are presented as mean ± SEM. p-values of the effects of NEB level at 1 WPP, time and interaction between NEB and time were considered significant if p-value < 0.05. With a,b and c the difference intra group and A, B the difference inter groups. Bold values represents significant values. ND, Not Determined.

### Energy balance, dry matter intake, live body weight, variation in empty body weight, milk yield and back fat thickness

One month before the presumed calving date, animals were kept in a place not equipped with defined feeders so it was not possible to determine **EB** during this period, thus, EB was only calculated from calving to 16WPP. The EB was calculated per week according to the INRA feeding system (INRA, 2007) as the difference between energy intake and energy requirements for maintenance and milk production. The dry matter intake (DMI) was estimated from the DM content of the feed and the intake of fresh matter as measured automatically through a unique passive transponder attached to the ear tag and automatic feeders (RIC software, version RW1.7, Incentec, Marknesse, the Netherlands). Cows were milked twice a day and were automatically weighed after each milking (software RIC version RW1.7, Hokofarm Group, Marknesse, The Netherland), and milk yield (MY) of each cow was automatically recorded through electronic collars (software R-Manufeed 500 pro, vc5 version 2.011.14, 1996, Manus-Delaval, Elancourt, France). Energy-corrected milk (ECM, 4% fat, 3.3% protein) was calculated according to the following equation: ECM = (milk production × 0.383 × fat % + milk production × 0.242 × protein % + 0.7832 × milk production/3.1138). Live body weight (LBW) was measured only during the morning milking to avoid the variation of body weight that occurs during the afternoon. LBW is affected by digestive contents so empty body weight (EBW) was estimated and corrected for digestive tract content, as described in Mellouk et al. (13). Adipose tissue mobilization was assessed through subcutaneous fat thickness measurements in the sacral region using ultrasonographic examination with a linear probe (LA 332 3.5/10.0-MHz transducer; Mylab30vet; R-Easote, Hospimedi, Saint-Crépin-Ibouvillers, France). Back fat thickness (**BFT**) was measured 4 WAP, 1 WPP and 16 WPP.

### Plasma biochemical parameters

Blood samples were collected from the coccygeal vein into heparinized vacutainers (Dutcher, Brumath, France) at 4WAP and 1 and 16 WPP (Figure 1). Blood was immediately centrifugated at 2,000 g for 15 min at 4°C and plasma was stored at−20°C until assay. NEFA, BHBA and glucose plasma concentrations were determined by enzymatic colorimetry assays (Wako Chemicals GmBH, Neuss, Germany; Randox Laboratories Ltd., Antrim, United Kingdom and Sigma Aldrich, Saint-Quentin-Fallavier, France, respectively). The intra and interassay coefficients of variation of plasma NEFA, BHBA and glucose measures were 6 and 7.8%, 4 and 6%, and 6 and 8%, respectively. Plasma insulin was measured by radio immuno-assay (RIA) from 100μL of undiluted plasma as described in Mellouk et al. (13).

Plasma bovine resistin, total adiponectin and leptin concentrations were determined with commercial bovine ELISA kits according to the manufacturer's protocol as described by Mellouk et al. (13). The sensitivity and the coefficient of variation of each ELISA kit used are indicated in Table 2.

**Table 2:** List of ELISA assays used for measurement of bovine plasma FNDC concentration.

**Protein**	**Supplier**	**Species**	**Assay sensitivity (ng/ml)**	**Coefficient of variation (%)**
Resistin	USCNLife/CLOUD Clone Corps. (E90847Bo)	Bovine	0.031	6.0
Total adiponectin	Hölzel (E11A0125)	Bovine	0.1	<8.0
Leptin	Hölzel (E11L0038)	Bovine	0.1	<8.0
FNDC3A	MyBioSource (#cat. MBS7201899-96)	Bovine	1.0	<10
FNDC3B	MyBioSource (#cat. MBS7211809-96)	Bovine	1.0	<10
FNDC4	MyBioSource (#cat. MBS7221653-96)	Bovine	0.1	<10
FNDC5	MyBioSource (#cat. MBS7246743-96)	Bovine	0.1	<10

Commercially available bovine ELISA kits (Table 2) were used to quantify plasma concentrations of FNDC3A, FNDC3B, FNDC4 and FNDC5 at 4WAP, 1 WPP and 16 WPP. Samples were run in duplicate within the same assay. The sensitivity and the coefficient of variation of each ELISA kit used are indicated in Table 2.

### Adipose tissue biopsies

Subcutaneous adipose tissue biopsies were carried out 4WAP, 1 WPP and 16 WPP. Cows were fasted for 12 h before surgery and sedation was induced by intravenous injection of 12–14 mg of Xylazine (Rompun^®^, Bayer, Leverkusen, Germany) and subcutaneous injection of 20 mg of Lidocaine (Lurocaïne^®^, Vetoquinol, Lure, France). Subcutaneous adipose tissue was collected from the dewlap under the neck, immediately frozen in liquid nitrogen and stored at −80°C until assay.

### RNA extraction and RT-qPCR

Total RNA was extracted on ice from 30 mg of tissue with an Ultraturax T25 Basic IKA Labortechnik homogenizer (Amilabo, Chassieu, France) in 1 ml of QIAzol lysis reagent (Qiagen, Courtaboeuf, France). One hundred microliter of chloroform (VWR chemicals, France) were added to each lysate. The samples were vortexed for 10 seconds then centrifuged for 15 min at 13,300 rpm at 4°C. The aqueous phase was recovered and then mixed with 500 μl of isopropanol (Carlo Erba Reagent, France), and placed on ice for 10 min before centrifugation (10 min at 13,300 rpm at 4°C). The supernatant was removed and the pellet was resuspended in 1 ml of 75% ethanol (diluted from absolute ethanol, Carlo Erba, France) then the samples were vortexed and centrifuged for 5 min at 8,800 rpm at 4°C. The supernatant was removed, and the pellet was air-dried, and resuspended in 40 μl of sterile demineralized water. DNAse treatment (NucleoSpin^®^ RNA Plus, Macherey-Nagel, France) was applied to the samples and the quantity and the quality of RNA was determined with a NanoDrop 2000 spectrophotometer (Thermo Scientific, USA).

The cDNAs were generated by reverse transcription (RT) of total RNA (1 μg) in a mixture with 0.5 mM of each deoxyribonucleotide triphosphate (dATP, dGTP, dCTP, dTTP), 2 M of RT buffer, 15 μg/μl of oligodT primer, 0.125 U of ribonuclease inhibitor (Recombinant RNasin^®^ Ribonuclease Inhibitor) and 0.05 U of M-MLV (Moloney Murine Leukemia Virus) Reverse Transcriptase (Promega, Charbonnières-Les-Bains, France) and incubated for 1 h at 37°C. The qPCR was carried out using the MyiQ Cycle device (Bio-Rad, Marnes-la-Coquette, France) in a mixture of SYBR Green Supermix 1X reagent (Bio-Rad, Marnes-la-Coquette, France), 250 nM specific primers (Invitrogen by Life Technologies, Villebon-sur-Yvette, France; Table 3) and sterile demineralized water forming a volume of 8 μl added to 3 μl of cDNA diluted 1:5 for a total volume of 20 μl. The samples were duplicated on the same plate. The control well had water instead of cDNA and was routinely performed as a negative control. After incubation for 2 min at 50°C and a denaturation step of 10 min at 95°C, the samples were subjected to 40 cycles (30 s at 95°C, 30 s at 60°C, 30 s at 72°C) followed by the acquisition of the melting curve. The efficiency (*E*) of the primers was determined from a serial dilution of cDNA and was calculated as *E* = 10^−1/*slopevalue*^, and was between 1.9 and 2.2 (Table 3). For each gene, relative abundance was calculated according to primer efficiency (*E*) and quantification cycle (Cq), where expression = *E*^−Cq^. Melting curve analysis were performed to verify the presence of a single amplicon. The expression of the target gene was expressed relative to the geometric mean of three specific reference genes, peptidylprolyl isomerase A (*PPIA*), succinate dehydrogenase complex flavoprotein subunit A (*SDHA*), and ubiquitously expressed prefoldin like chaperone (*UXT*) previously validated and that were not affected by the physiological status and the EB level (42, 43). Same housekeeping genes were used for all samples except for *HSL* and *ATGL* where *Cyclophilin A* (*PPIA*), *GAPDH* and β*-actin* were used as reference gene. The geometric mean of housekeeping genes has been reported as an accurate normalization factor (43).

**Table 3:** Nucleotide primer sequences used for real time RT-PCR^a^.

**Gene**	**Species**	**Product length (pb)**	**Forward**	**Reverse**	**Number of accession**	**Efficiency**
*PPIA*	bov	216	5^′^-GCATACAGGTCCTGGCATCT-3^′^	5^′^-TGTCCACAGTCAGCAATGGT-3^′^	NM_178320.2	1.9
*PPIA*	hum	161	5^′^-GGCAAATGCTGGACCCAACACA-3^′^	5^′^-TGCTGGTCTTGCCATTCCTGGA-3^′^	NM_021130	2.0
*SDHA*	bov	168	5^′^-TATATGGCGCTGGCTGTCTC-3^′^	5^′^-CCTCTTCCCTCGCGGATTTC-3^′^	NM_174178.2	2.0
*SDHA*	hum	279	5^′^-GAAGGTCTCTGCGATATGA-3^′^	5^′^-CTGTAGGGTGGAACTGAAC-3^′^	NM_004168.4	1.9
*UXT*	bov	111	5^′^-CATTGAGCGACTCCAGGAAG-3^′^	5^′^-GGCCACATAGATCCGTGAAG-3^′^	NM_001037471.2	1.9
*UXT*	hum	80	5^′^-AGTGCTGCGCTACGAGACCT-3^′^	5^′^-ATACCTTGTCTCGATGGTCC-3^′^	NM_153477.3	1.8
*FNDC3A*	bov	99	5^′^-TAAGTGCAGATGGAACACAACAG-3^′^	5^′^-CTGTAATGCACTGAAACTGTCCA-3^′^	NM_001205646.1	2.0
*FNDC3B*	bov	135	5^′^ATGCCTCACTTGGTGAATGGAG-3^′^	5^′^-CATCATGGGAACTTCAGCAGGT-3^′^	NM_001206211.2	2.0
*FNDC4*	bov	148	5^′^-GGCAACGTGTGATCCGAGAG-3^′^	5^′^-AGAGTTCGGAAGTGCACCCT-3^′^	NM_001102324.1	2.0
*FNDC5*	bov	187	5^′^-GGTAAGCTGGGATGTCTTGG-3^′^	5^′^-CTGACCCTGGATGGATATGG-3^′^	NM_001105421.1	2.0
*ITGAV*	bov	183	5^′^-TTCGGCTATTCCATGAAAGG-3^′^	5^′^-AGGCAGAGGGCAGGTTTTAT-3^′^	NM_174367.1	2.0
*ITGB1*	bov	231	5^′^-TGAGGTGAACAGCGAAGACA-3^′^	5^′^-TTGCACTCACACACTCGACA-3^′^	NM_174368.3	2.0
*ADGRF5*	bov	189	5^′^-TATCCTGAGAATGTCGGTCAGACT-3^′^	5^′^-TCGTACGTCACAACCACACT-3^′^	NM_001193243.1	2.0
*ATGL*	bov	130	5^′^-AAGCTGGTGCCAACATCATC-3^′^	5^′^-TAGCAATCAGCAGGCAGAAT-3^′^	NM_001046005.2	2.0
*HSL*	bov	199	5^′^-GAGACTGGCATCAGTGTGAC-3^′^	5^′^-TTGCTAGAGACGATAGCACCT-3^′^	NM_001080220.1	2.0
*ATGL*	hum	119	5^′^-GTGTCAGACGGCGAGAATG-3^′^	5^′^-TGGAGGGAGGGAGGGATG-3^′^	NM_020376.4	2.0
*HSL*	hum	298	5^′^-GTGCAAAGACGGAGGACCACTCCA-3^′^	5^′^-GACGTCTCGGAGTTTCCCCTCAG-3^′^	NM_005357.4	1.9

^a^hum, human; bov, bovine.

To survey FNDC mRNA abundance in a variety of tissues, samples of bovine pituitary, kidney, skeletal semitendinosus muscle, liver, heart, and subcutaneous adipose tissue were collected from a local slaughterhouse within 30 min after sacrifice and frozen in liquid nitrogen before extraction of RNA.

### Protein extraction and western blot

Tissues were lysed using Ultraturax T25 Basic IKA Labortechnik (Amilabo, Chassieu, France) in lysis buffer [1 M Tris (pH 7.4), 0.15 M NaCl, 1.3 mM EDTA, 1 mM EGTA, 23 mM $VO_{4}^{3}$, 0.1 M NaF, 1% NH_2_PO_4_, 0.5% Triton). The lysates were centrifuged for 20 min at 13,300 rpm at 4°C and the supernatant containing the proteins was collected and stored on ice. Protein concentration was measured using the Bicinchoninic Acid Protein Assay (BCA; Interchim, Montluçon, France). The protein lysates (40 μg) were mixed with 5X Laemmli buffer and the proteins were denatured for 5 min by heat shock at 95°C. The proteins were loaded into a 15% sodium dodecyl sulfate-polyacrylamide gel, and after electrophoresis the proteins were transferred from the gel to a nitrocellulose membrane. The membranes were blocked with 0.05% Tween 20 and 5% powdered cow's milk in Tris-saline buffer for 30 min at room temperature. The membranes were then incubated overnight at 4°C with the appropriate primary antibody (Table 4). The anti-irisin (12.6 kDa) antibody also recognizes FNDC5 with a molecular weight of ~25 kDa (44). The membranes were then incubated for 90 min at room temperature with a HorseRadish Peroxidase-conjugated (HRP) anti-rabbit IgG secondary antibody (Table 4). The proteins of interest were detected by enhanced chemiluminescence (Western Lightning Plus-ECL, Perkin Elmer, Yvelines, France), with a G: Box Syngene (Ozyme) and GeneSnap software [(version 7.09.17), Ozyme]. Signal intensity was quantified with the GeneTools software, and the results expressed relative to β-actin or vinculin.

**Table 4:** List of antibodies used for the detection of proteins by Western blot.

**Primary antibodies**	**Dilution**	**Supplier**	**Host**	**Molecular weight** **(kDa)**
β-actin	1:1000	Sigma	Rabbit	42
Irisin (42–112)	1:1000	Phoenix Pharmaceuticals, Inc	Rabbit	25 and 12.6
Vinculin	1:200	Sigma-Aldrich	Mouse	110
Secondary antibodies
Anti-mouse	1:5000	Millipore	Goat	
Anti-rabbit	1:5000	Biorad	Goat	

### Adipose tissue explant culture

Abdominal adipose tissue was collected from 6 dairy cows at a local slaughterhouse immediately after death, and was transported to the laboratory at 37°C in DMEM supplemented L-glutamine (PAA Laboratories GmbH, Cölbe, Germany). For each animal, the tissue (200 mg) was cut into 10 small pieces under sterile conditions and incubated in quadruplicate with 250 μl of DMEM supplemented with penicillin/streptomycin and amphotericin with increasing doses of human recombinant irisin (0, 0.1, 1 and 10 ng/ml) for 24 h at 37°C under an atmosphere containing 5% CO_2_. The tissue explants and the culture medium were then collected and immediately stored at −20°C.

Human abdominal subcutaneous adipose tissue was collected from patients (*n* = 6) undergoing bariatric surgery and included in the prospective monocentric METABOSE cohort (Nutrition Department, CHU Tours) following patient written consent and after local ethical committee agreement (CNIL no. 18254562). Once collected, adipose tissue explants were prepared and were cultured under the same conditions as bovine adipose tissue explants as described above.

### *In vitro* lipolysis assay

The rate of lipolysis in the adipose tissue explants was determined by measuring glycerol release into the incubation medium (Sigma, St. Louis, MO, USA) and by quantifying abundance of mRNA encoding *HSL* and *ATGL*.

### Statistical analysis

All statistical analyses were performed with GraphPad Prism 6 software. Data were tested for homogeneity of variance by Bartlett's test and for normal distribution by Shapiro-Wilk test. A two-way analysis of variance (ANOVA) with repeated measures was carried out to determine effects of time and NEB level at 1 WPP (LNEB vs. HNEB) on endpoints measured. Furthermore, the interaction, NEB level at WPP and the time was also analyzed. A Sidak correction was applied to multiple comparisons. A one-way analysis of variance (ANOVA) was carried out to analyse gene and protein expression in metabolic tissues and lipolysis. A Tukey correction was applied to multiple comparisons. A unidirectional non-parametric analysis of variance (Friedman test) with repeated measures was carried out to analyse plasma FNDC concentrations and protein expression in adipose tissue in each group and gene expression in adipose tissue of both groups. A Dunn correction was applied to multiple comparisons. The correlation between physiological (zootechnical, plasma metabolites and metabolic hormones) parameters and FNDC mRNA abundance with plasma concentrations were investigated by using Spearman correlations. All experimental data are presented as means ± SEM. The level of statistical significance was set at *p* < 0.05.

## Results

### Energy balance, live body weight, variation empty in body weight, milk yield, energy corrected milk, dry matter intake and fat back thickness

Live body weight, variation of empty body weight (VEBW), and back fat thickness (BFT) were measured at 4 WAP, 1 and 16 WPP and energy balance (EB), dry matter intake (DMI), milk yield (MY) and fat and protein energy corrected milk (ECM) were measured at 1 and 16 WPP in both LNEB and HNEB animals. As described above, LNEB and HNEB groups were determined according to the EB at 1 WPP (−5.60 ± 0.77 Mcal/day for LNEB and −12.16 ± 0.53 Mcal/day for HNEB) and to their plasma BHBA concentrations at 1 WPP (mean ± SEM for LNEB and HNEB group: 1.16 ± 0.11 mmol/L and 1.31 ± 0.03 mmol/L, respectively; Table 1). For all the zootechnical parameters studied except for protein ECM, we observed a significant effect of peripartum time and a significant difference between LNEB and HNEB animals except for MY, fat ECM and VEBW (Table 1).

### Plasma glucose, NEFA, BHBA and insulin

To assess mobilization of adipose tissue and ketogenesis, plasma NEFA and BHBA were measured, respectively. There was a significant effect of NEB status at 1WPP on BHBA but not on NEFA plasma concentrations. However, there was a significant time effect with an increase of NEFA and BHBA plasma concentrations at 1 WPP as compared to 4 WAP for both HNEB and LNEB animals and at 1 WPP as compared with 16 WPP for only HNEB animals. At 1WPP, BHBA plasma concentrations were higher in HNEB than in LNEB animals (Table 1). Plasma glucose concentrations were affected by peripartum time only in HNEB group with a decrease at 1 WPP as compared with 4 WAP and 16 WPP (Table 1). There was no significant effect of either NEB status at 1 WPP or peripartum time on plasma insulin concentrations.

### Plasma concentration of adiponectin, leptin and resistin

Plasma concentrations of adiponectin, leptin and resistin were significantly affected by both NEB level at 1 WPP and peripartum time (Table 1). Plasma leptin concentrations decreased at 1 and 16 WPP as compared to 4WAP, and were significantly lower in HNEB group than in LNEB group post-partum. Plasma adiponectin concentrations decreased at 1WPP compared to 4WAP and increased at 16WPP to levels not different from 4WAP; adiponectin concentrations were higher in LNEB than in HNEB animals at 16WPP. Plasma resistin concentrations increased at 1 WPP as compared to 4 WAP, and then declined at 16WPP to levels below those at 1WAP. At 1WPP, resistin concentrations were higher in HNEB compared to LNEB animals (Table 1).

### Plasma concentration of FNDCs and association of plasma FNDC5 concentrations with other metabolic parameters

**Figure 2:**
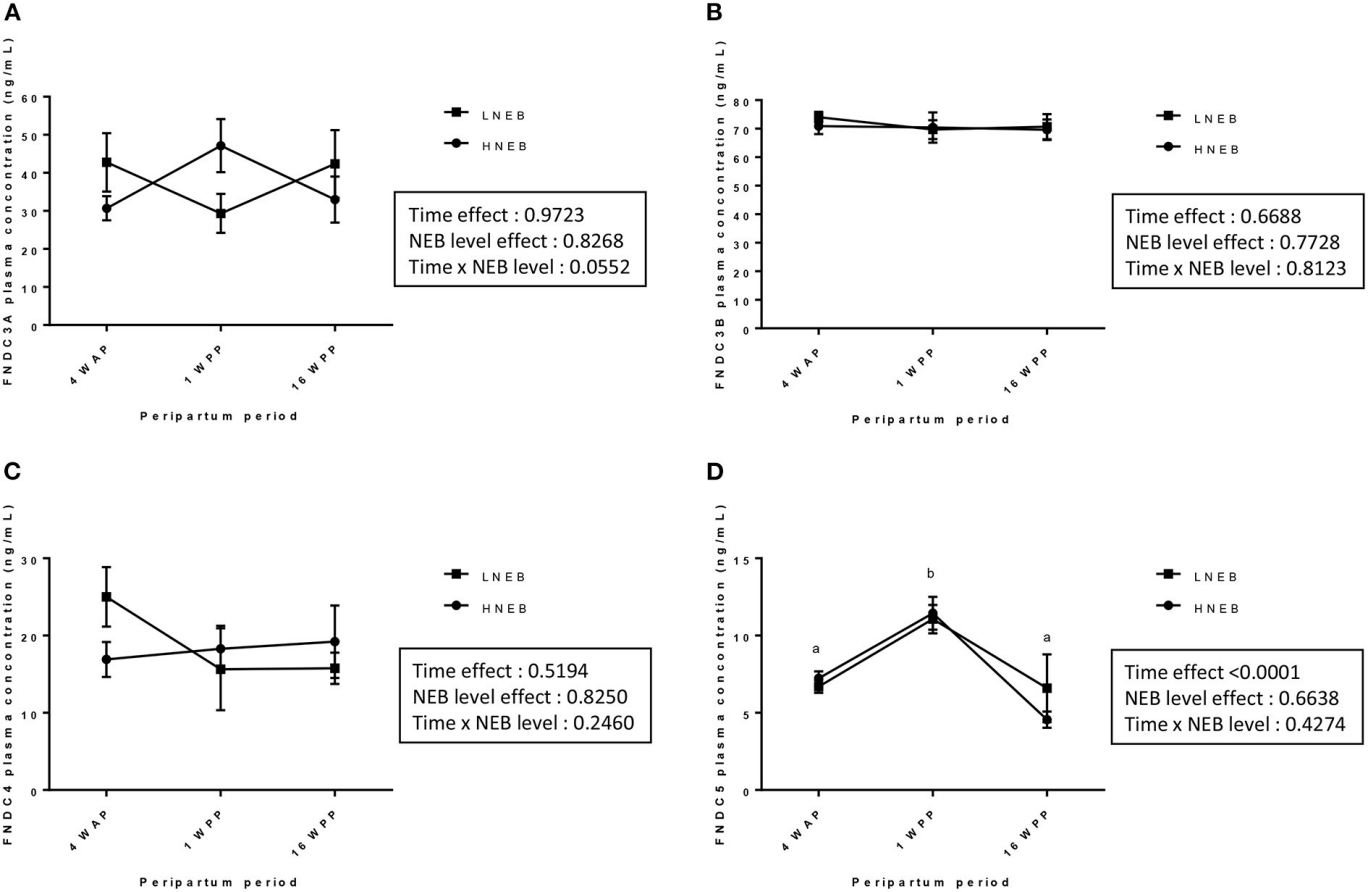
Plasma concentrations of FNDC proteins at 4 WAP, 1 WPP and 16 WPP in HNEB and LNEB animals. The concentrations of FNDC3A **(A)**, FNDC3B **(B)**, FNDC4 **(C)** and FNDC5 **(D)** in plasma were determined by ELISA assays. Data are expressed as mean ± SEM for each group of animals (HNEB and LNEB) at each stage of the peripartum period (4WAP, 1 and 16 WPP, *n* = 8) and analysed by a nonparametric analysis of variance with repeated measures (Friedman test). Groups with different letters were significantly different (*p*-value < 0.05).

By using specific bovine ELISA assays, we investigated plasma concentration of FNDC3A, FNDC3B, FNDC4 and FNDC5 at 4 WAP, and at 1 and 16 WPP in LNEB and HNEB animals. We did not observe any difference between LNEB and HNEB animals for all FNDCs. There were no significant changes in FNDC3A, FNDC3B and FNDC4 concentrations during the peripartum period, whereas plasma FNDC5 concentrations were significantly higher at 1 WPP compared with 4 WAP and 16 WPP in both group (*p*-value: 0.0179 in LNEB group and *p*-value: < 0.0001 in HNEB group; Figure 2 and Table 5).

**Table 5:** Plasma FNDC3A, FNDC3B, FNDC4 and FNDC5 concentrations in HNEB and LNEB animals at 4 WAP and 1 and 16 WPP.

**FNDC**	**HNEB**			**LNEB**			***p*** **-Value**		
	**4 WAP**	**1 WPP**	**16 WPP**	**4 WAP**	**1 WPP**	**16 WPP**			
	**Mean** ±**SEM**	**Mean** ±**SEM**	**Mean** ±**SEM**	**Mean** ±**SEM**	**Mean** ±**SEM**	**Mean** ±**SEM**	**NEB at 1 WPP**	**Time**	**NEB level x Time**
FNDC3A (ng/ml)	30.68 ± 3.39	47.14 ± 7.45	32.97 ± 6.49	42.75 ± 8.21	29.35 ± 5.48	42.36 ± 9.49	0.83	0.97	0.06
FNDC3B (ng/ml)	70.92 ± 3.04	70.41 ± 5.65	69.63 ± 3.87	74.03 ± 1.97	69.68 ± 3.54	70.70 ± 4.69	0.77	0.67	0.81
FNDC4 (ng/ml)	16.90 ± 2.45	18.29 ± 3.22	19.20 ± 5.06	25.01 ± 4.15	15.65 ± 5.72	15.76 ± 2.20	0.83	0.52	0.25
FNDC5 (ng/ml)	7.23^ab^ ± 0.51	11.44^b^ ± 1.14	4.55^a^ ± 0.52	7.04^ab^ ± 0.39	10.73^b^ ± 0.92	6.58^a^ ± 2.19	0.66	<0.0001	0.43

Data are mean ± SEM and were analyzed by two-way analysis of variance (ANOVA). The results were considered significant if p-value < 0.05. Bold values represents significant values. Groups with different letters are significantly different (p < 0.05).

We next determined potential correlations between plasma FNDC5 concentrations and zootechnical parameters, plasma metabolites and hormones by combining data from HNEB and LNEB animals during the period 4WAP to 16 WPP. As shown in Table 6, plasma FNDC5 concentrations were negatively correlated with DMI, LBW, VEBW, glucose and insulin levels, and were positively associated with plasma NEFA, BHBA, leptin and adiponectin concentrations.

**Table 6:** Spearman correlation coefficients (r) between FNDC5 plasma concentration and zootechnical parameters and plasma metabolites and hormones in both LNEB and HNEB animals during the peripartum period (4WAP−16WPP).

**Parameter**		**Plasma FNDC5**
Energy balance (EB, Mcal/day)	*r*	−0.252
	*p*-value	0.165
Dry matter intake (DMI, kg/day)	*r*	–0.359
	*p*-value	0.044
Live body weight (LBW, kg/day)	*r*	–0.364
	*p*-value	0.012
Variation of empty body weight	*r*	–0.496
(VEBW, kg/day)	*p*-value	3.9 *10^−4^
Milk Yield (MY, kg/day)	*r*	−0.126
	*p*-value	0.491
Back fat thickness (BFT, cm)	*r*	−0.137
	*p*-value	0.360
Fat energy corrected milk (kg/day)	*r*	0.029
	*p*–value	0.875
Protein energy corrected milk (kg/day)	*r*	−0.033
	*p*-value	0.856
Glucose (mmol/L)	*r*	–0.344
	*p*-value	0.018
NEFA (mmol/L)	*r*	0.610
	*p*-value	5.4 *10^−6^
BHBA (mmol/L)	*r*	0.731
	*p*-value	7.9 *10^−9^
Insulin (ng/ml)	*r*	–0.696
	*p*-value	3.4 *10^−6^
Leptin (ng/ml)	*r*	0.349
	*p*-value	0.027
Adiponectin (μg/ml)	*r*	0.655
	*p*-value	7.8 *10^−7^
Resistin (ng/ml)	*r*	0.162
	*p*-value	0.288

Correlations were considered significant if p-value < 0.05. Bold values represents significant values.

### FNDC and receptor expression in tissues

**Figure 3:**
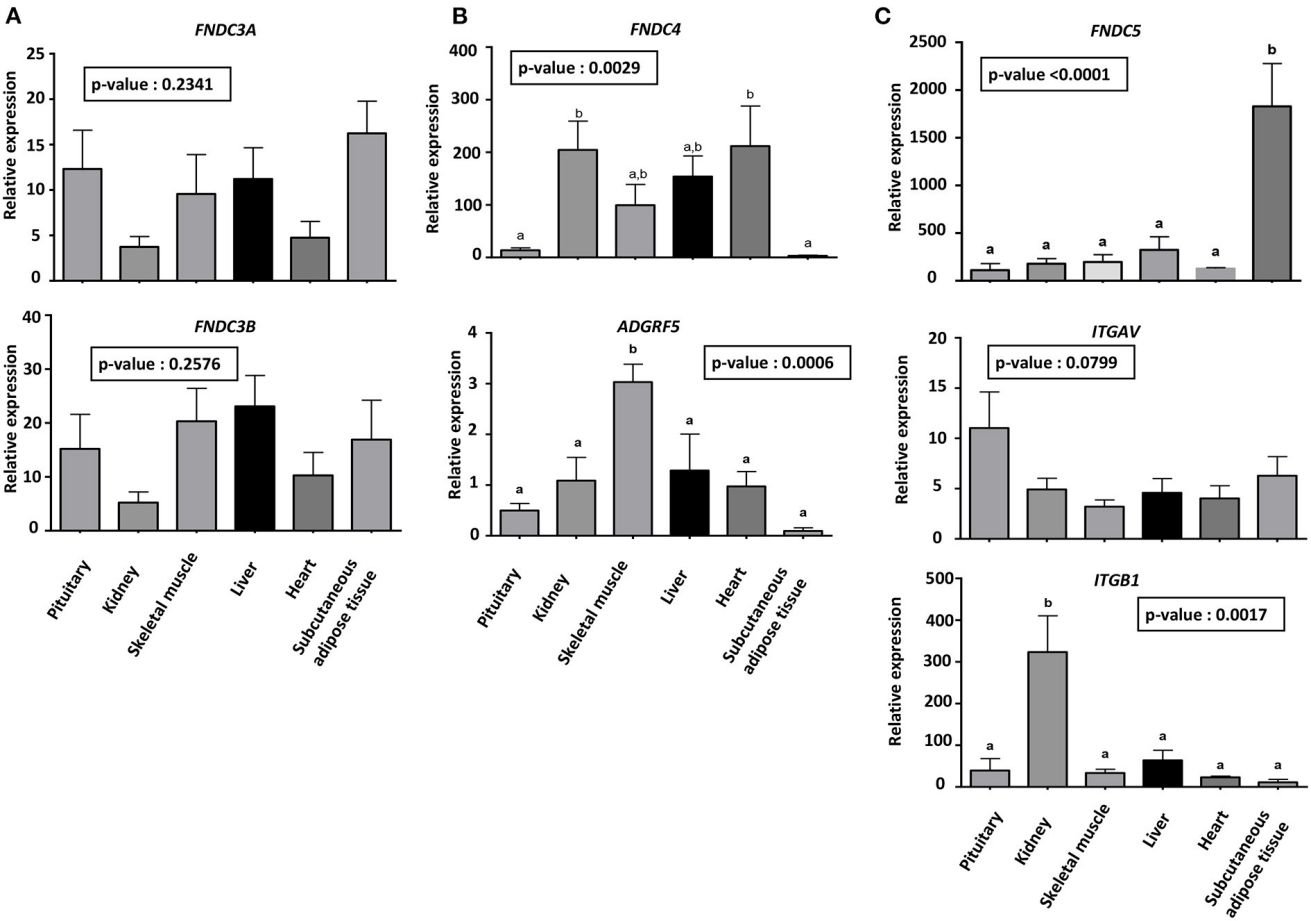
Abundance of mRNA encoding FNDCs, ADGRF5, ITGAV and ITGB1 **(A–C)** in various bovine tissues. Abundance of mRNA was determined by real-time RT-qPCR. Data are expressed as mean ± SEM (n = 8 animals) and analysed by a one-way analysis of variance (ANOVA). Different letters indicate a significant difference between tissues (*p* < 0.05).

We next investigated gene expression of FNDCs and FNDC4 and five receptors (*ADGRF5, ITGAV* and *PITGB1*) at the mRNA level in various bovine tissues. As shown in Figure 3, abundance of *FNDC3A, FNDC3B* and *ITGAV* mRNA was similar in all tissues studied. The abundance of *ADGRF5* mRNA was higher in skeletal muscle compared with the other tissues, and *ITG1B* mRNA was most abundant in the kidney (*p* < 0.05). Messenger RNA encoding *FNDC5* was most abundant in subcutaneous adipose tissue as compared to the other tissues and mRNA encoding *FNDC4* was more expressed in kidney and heart than in subcutaneous adipose tissue and pituitary.

**Figure 4:**
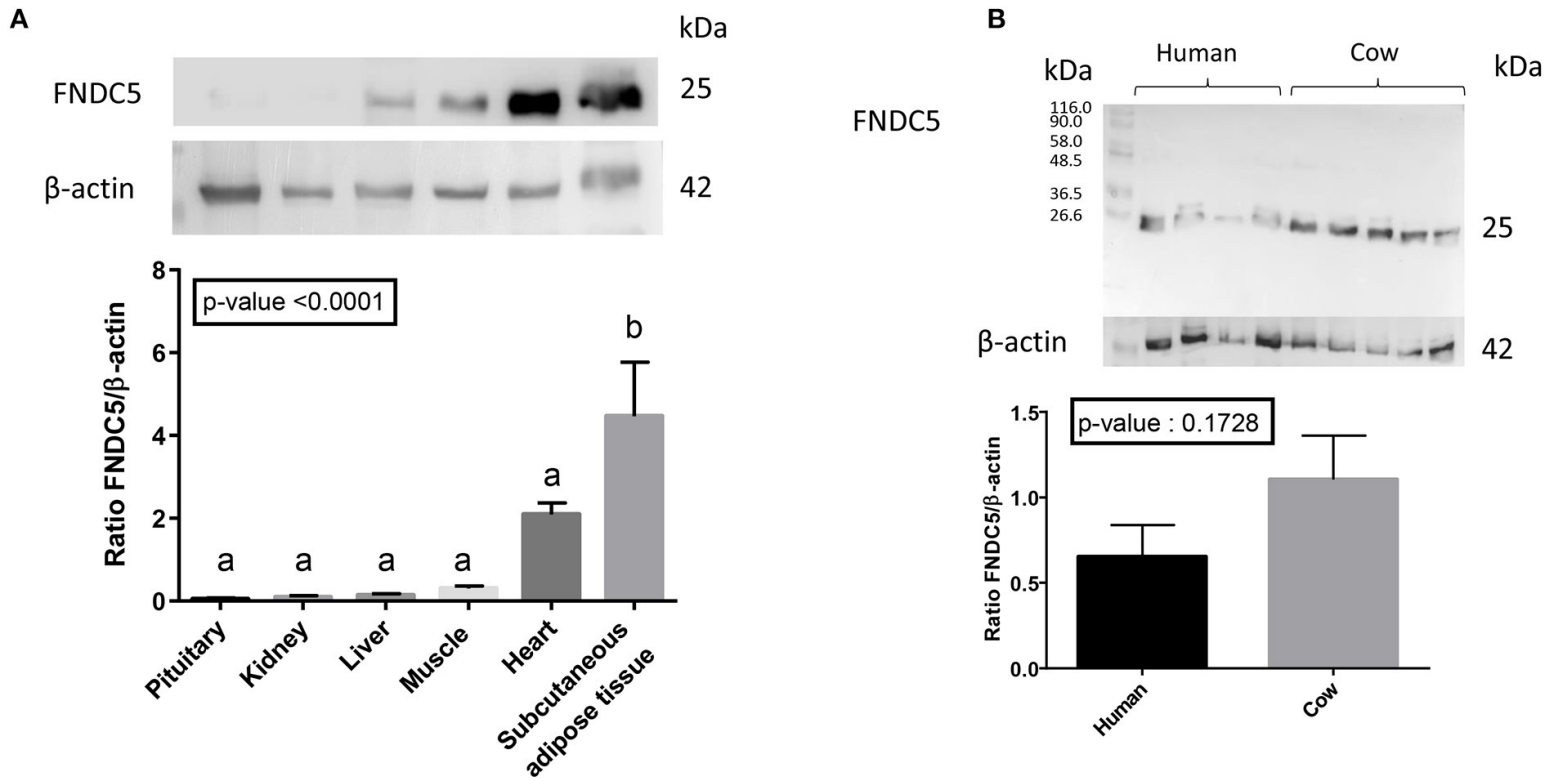
Protein expression of FNDC5 in different bovine tissues. **(A)** Abundance of FNDC5 protein was determined by western blot in tissues obtained from an abattoir. A representative blot is shown above the means. Data are expressed as mean ± SEM for each group (*n* = 7) and analyzed by one-way analysis of variance (ANOVA). **(B)** Bovine (*n* = 5) and human (*n* = 4) abdominal subcutaneous adipose tissues were examined for full-length (25 kDa) and cleaved (12 kDa) forms of FNDC5/irisin.

Using a commercial human anti-irisin antibody, we confirmed the higher expression of FNDC5 protein (25 kDa) in bovine subcutaneous adipose tissue and heart compared with pituitary and kidney (Figure 4A). This antibody did not detect cleaved irisin (expected mass 12.6 kDa) in bovine or human adipose tissue (Figure 4B).

### FNDCs and receptor expression in subcutaneous bovine adipose tissue during peripartum in LNEB and HNEB animals

**Figure 5:**
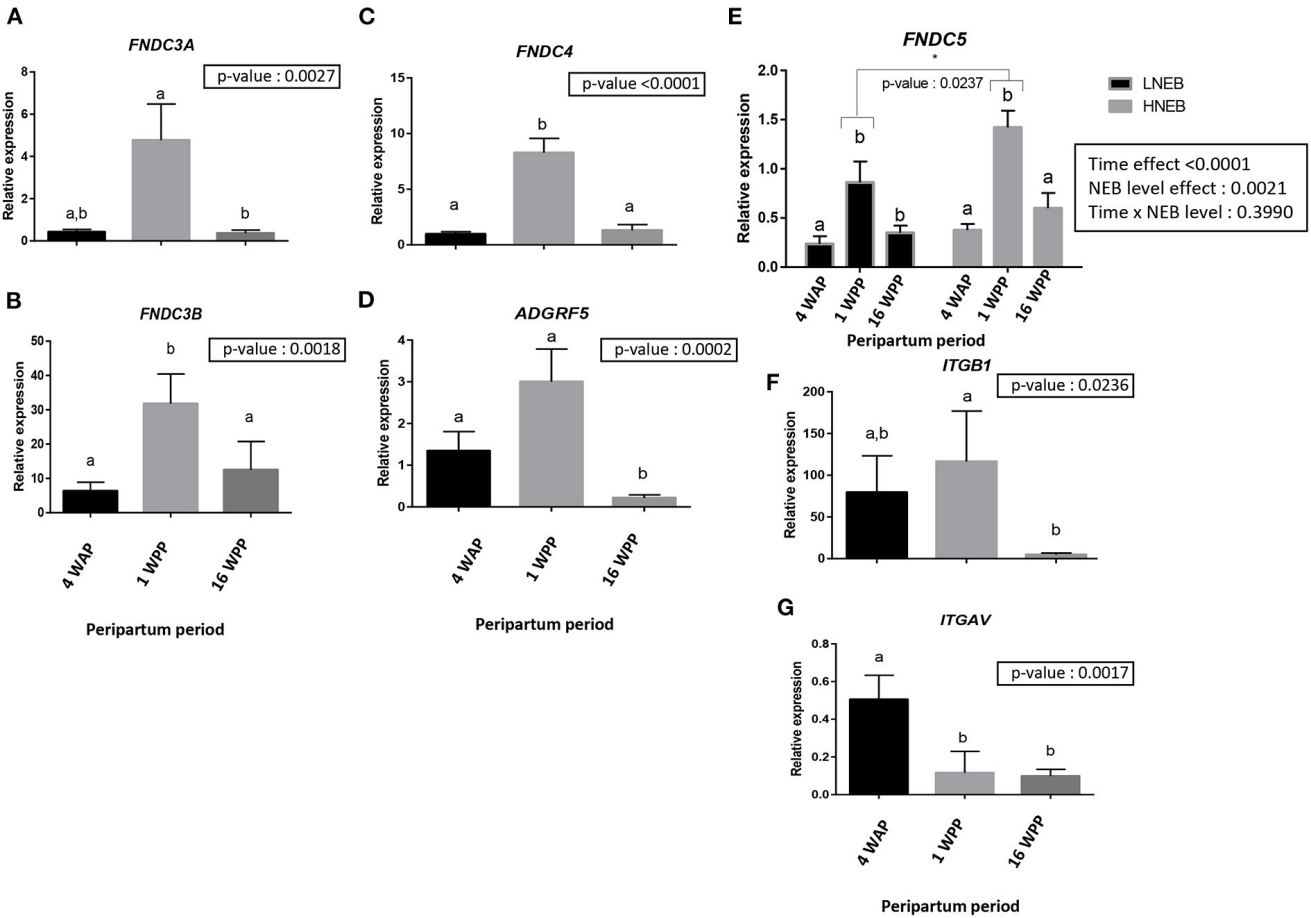
Abundance of FNDC3A **(A)**, FNDC3B **(B)**, FNDC4 **(C)**, FNDC5 **(E)**, ITGAV **(G)**, ITGB1 **(F)** and ADGRF5 **(D)** mRNA in bovine subcutaneous adipose tissue during peripartum period. Tissues were obtained 4 weeks before parturition (4WAP) and 1 (1WPP) and 16 weeks post-partum (16 WPP) and mRNA measured by real-time RT-qPCR. Data are expressed as mean ± SEM for each group (*n* = 8 in LNEB group and *n* = 13 in HNEB group) and analysed by a nonparametric analysis of variance with repeated measures (Friedman test). Groups with different letters are significantly different (*p* < 0.05). * indicates a significant difference (*p* < 0.05).

We next determined gene expression of *FNDC3A, FNDC3B, FNDC4, FNDC5, ADGRF5, ITGAV* and *ITGB1* in subcutaneous adipose tissue biopsies at 4 WAP, 1 WPP and 16 WPP in LNEB and HNEB animals. There was no significant difference between LNEB and HNEB animals for all the genes studied except for *FNDC5* mRNA (Table 7), therefore we combined the two groups of animals for these genes and analyzed the effect of peripartum time (Figures 5A–G). Abundance of *FNDC3B* (Figure 5B) and *FNDC4* (Figure 5C) mRNA was significantly higher at 1WPP as compared with both 4WAP and 16 WPP, whereas *FNDC3A* (Figure 5A) and *ITGB1* (Figure 5F) mRNA abundance was significantly higher at 1WPP compared with 16WPP. The abundance of *ADGRF5* mRNA (Figure 5D) was lower at 16 WPP compared to both 4WAP and 1WPP whereas that of *ITGAV* (Figure 5G) was higher at 4WAP compared with 16WPP and 1WPP. Abundance of *FNDC5* mRNA was significantly increased at 1WPP compared to 4WAP and 16WPP in the HNEB group and significantly increased at 1WPP as compared to 4WAP only in the LNEB group. *FNDC5* mRNA abundance was higher in HNEB compared with LNEB animals 1WPP (Figure 5E, Table 7).

**Table 7:** Relative gene expression of *FNDC3A, FNDC3B, FNDC4, FNDC5, ITGAV, ITGB1* and *ADGRF5* in LNEB and HNEB animals during the peripartum period (1WAP, 1 and 16WPP)^1^.

**Gene**	**HNEB**			**LNEB**			***p*** **-Value**		
	**4 WAP**	**1 WPP**	**16 WPP**	**4 WAP**	**1 WPP**	**16 WPP**			
	**Mean** ±**SEM**	**Mean** ±**SEM**	**Mean** ±**SEM**	**Mean** ±**SEM**	**Mean** ±**SEM**	**Mean** ±**SEM**	**NEB at 1 WPP**	**Time**	**NEB level x Time**
*FNDC3A*	0.89^ab^ ± 0.45	4.28^a^ ± 1.79	0.17^b^ ± 0.06	0.34^ab^ ± 0.23	1.36^a^ ± 1.26	0.18^b^ ± 0.12	0.21	0.05	0.37
*FNDC3B*	2.93^a^ ± 1.24	38.15^b^ ± 13.85	7.38^a^ ± 4.42	2.57^a^ ± 1.9	24.44^b^ ± 15.35	0.76^a^ ± 0.48	0.35	0.004	0.75
*FNDC4*	1.03 ^a^ ± 0.29	8.39 ^b^ ± 1.55	1.88 ^a^ ± 0.82	0.76 ^a^ ± 0.32	8.11^b^ ± 2.66	0.38^a^ ± 0.09	0.81	<0.0001	0.55
*FNDC5*	0.38 ^aA^ ± 0.07	1.42 ^bA^ ± 0.18	0.60 ^aA^ ± 0.16	0.24^aA^ ± 0.08	0.86^bB^ ± 0.23	0.35^bA^ ± 0.08	0.002	<0.0001	0.40
*ITGAV*	0.44 ± 0.11	0.5 ± 0.34	0.11 ± 0.05	0.57^a^ ± 0.27	0.17^b^ ± 0.19	0.08^b^ ± 0.06	0.59	0.13	0.51
*ITGB1*	31.54 ± 18.21	35.86 ± 16.71	1.42 ± 0.56	7.31 ± 5.22	0.05 ± 0.01	1.51 ± 2.14	0.27	0.60	0.65
*ADGRF5*	1.08^a^ ± 0.28	3.73^a^ ± 1.15	0.32^b^ ± 0.12	0.94^a^ ± 0.87	0.52^a^ ± 0.37	0.25^b^ ± 0.25	0.08	0.03	0.03

Gene expression was determined by real-time RT-qPCR. Data are Mean ± SEM. The results were considered significant if p-value < 0.05.

^1^Results are presented as mean ± SEM. p-values of the effects of NEB level at 1 WPP, time and interaction between NEB and time were considered significant if p-value < 0.05. With a and b the difference intra group and A, B the difference inter groups. Bold values represents significant values.

**Figure 6:**
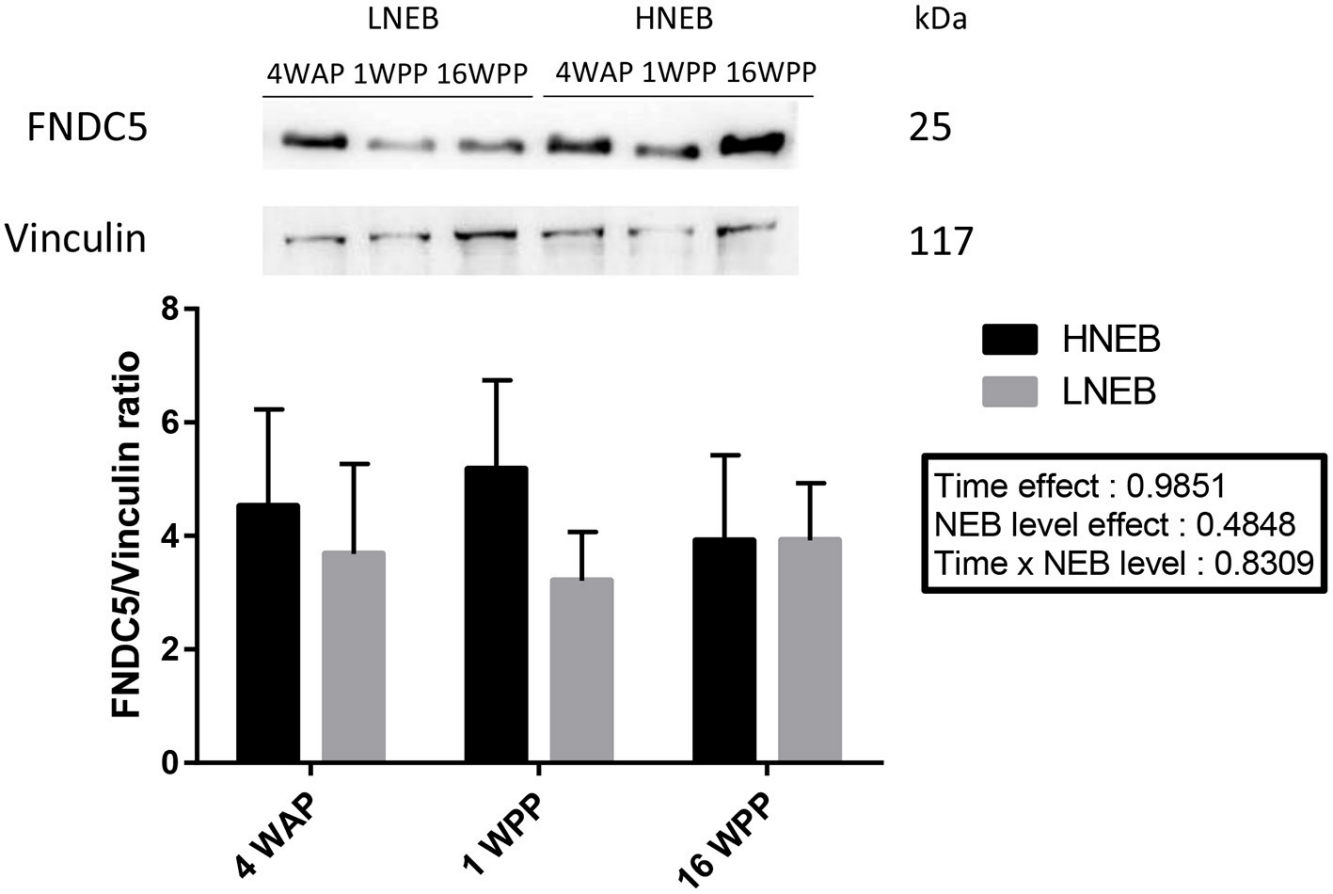
Protein expression of FNDC5 in bovine subcutaneous adipose tissue during peripartum period. Protein expression of FNDC5 was determined by western blot in tissues obtained 4 weeks before parturition (4WAP) and 1 (1WPP) and 16 weeks post-partum (16 WPP). A representative blot is shown above the means. Data are expressed as mean ± SEM for each group (*n* = 8 in LNEB group and *n* = 13 in HNEB group) and analyzed by a nonparametric analysis of variance with repeated measures (Friedman test).

We did not observe any variation of FNDC5 protein (25 kDa) abundance in bovine subcutaneous adipose tissue during the peripartum period or between NEB level (Figure 6).

We next determined potential correlations between adipose FNDC mRNA abundance and plasma FNDC concentrations. Only plasma FNDC5 concentrations was positively correlated with *FNDC5* mRNA abundance (Table 8). Plasma FNDC3A, FNDC3B and FNDC4 concentrations were not significantly correlated with their respective adipose tissue mRNA expression (Table 8).

**Table 8:** Spearman correlation coefficients (r) between FNDCs plasma concentration and FNDCs genes expression in adipose tissue by combining data from HNEB and LNEB animals during the peripartum period (4WAP−16WPP).

**Gene expression**		**Plasma FNDC3A**	**Plasma FNDC3B**	**Plasma FNDC4**	**Plasma FNDC5**
*FNDC3A*	*r*	−0.060			
	*p*-value	0.716			
*FNDC3B*	*r*		0.314		
	*p*-value		0.127		
*FNDC4*	*r*			−0.081	
	*p*-value			0.608	
*FNDC5*	*r*				0.384
	*p*-value				0.016

Correlations were considered significant if p-value < 0.05. Bold values represents significant values.

### Effect of recombinant human irisin on the release of glycerol and on adipose triglyceride lipase (ATGL) and hormone-sensitive lipase (HSL) mRNA abundance in bovine and human subcutaneous adipose tissue explants

**Figure 7:**
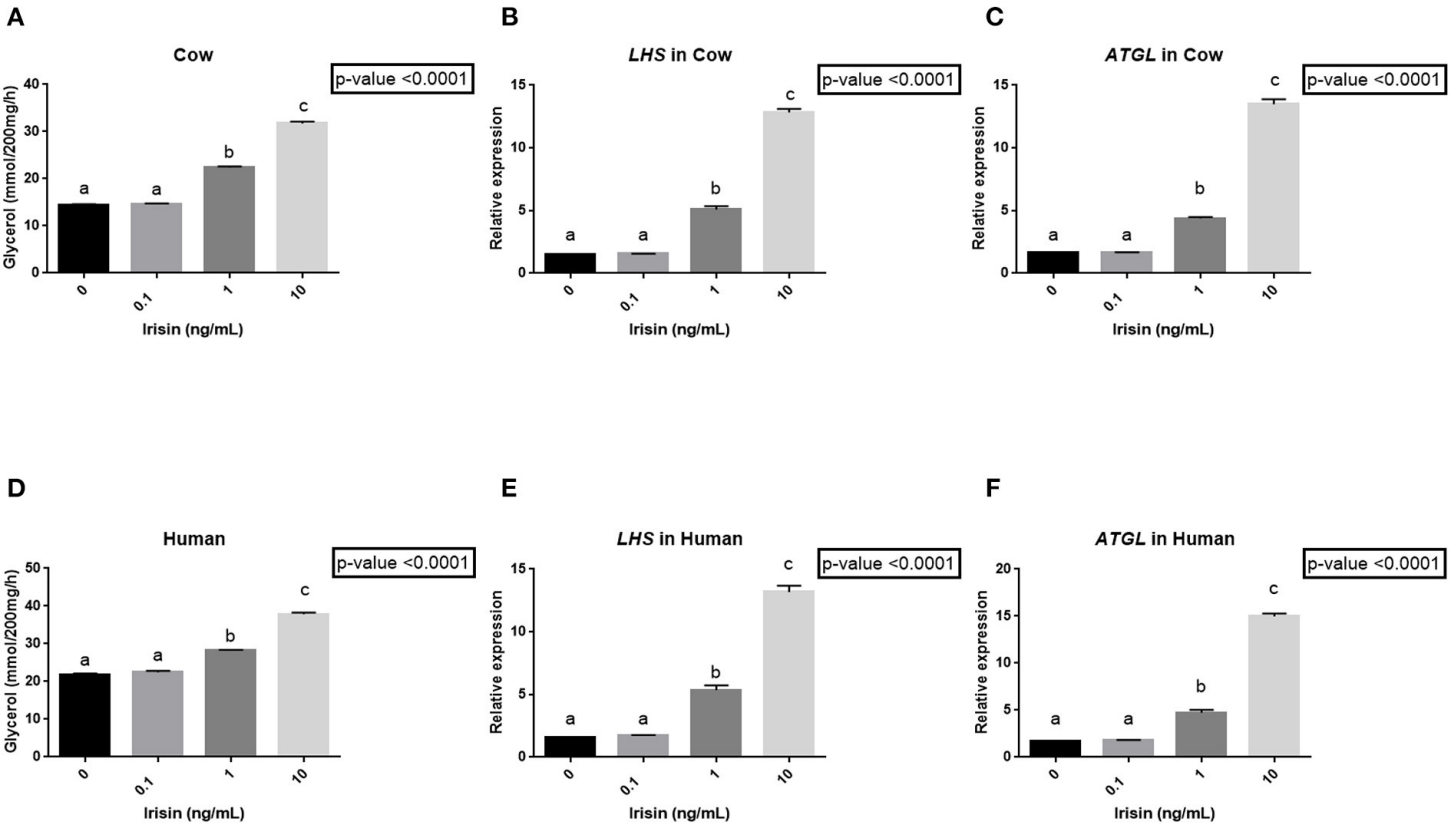
Effect of human recombinant irisin on the release of glycerol and on *HSL* and *ATGL* mRNA abundance from bovine **(A–C)** and human adipose tissue explants [**(D–F)**, *n* = 6 for each species]. **(A,D)** Data are expressed as millimoles per 200 mg of tissue per hour ± SEM and different letters indicate significant difference at *p* < 0.0001. **(B,E)** *ATGL* mRNA and **(C,F)** *HSL* mRNA abundance in adipose tissue explants incubated with increasing doses of human recombinant irisin (0; 0.1; 1 and 10ng/ml; *n* = 6). Abundance is expressed relative to the geometric mean of 3 reference genes [*Cyclophilin A* (*PPIA*), *GAPDH* and β*-actin*] and analyzed by one way analysis of variance (ANOVA). Data are presented as mean ± SEM. Different letters indicate significant differences at *p* < 0.0001.

Since plasma FNDC5 concentrations and adipose *FNDC5* mRNA abundance were higher at 1 WPP when the adipose tissue mobilization was greatest, we determined whether irisin could be involved in adipose tissue lipolysis. Human recombinant irisin significantly increased glycerol release (Figure 7A) and *HSL* (Figure 7B) and *ATGL* (Figure 7C) mRNA abundance in a dose-dependent manner in both bovine and human adipose tissue explants (Figures 7D–F).

## Discussion

This study provides several lines of evidence that the myokine/adipokine FNDC5/irisin plays a role in adipose tissue mobilization during post-partum negative energy balance in dairy cattle. First, both plasma concentrations of *FNDC5*/Irisin and *FNDC5* gene expression in adipose tissue increased 1 week after calving during NEB, compared to 4 weeks before and 16 weeks after calving. Second, recombinant irisin, which is the cleaved and secreted fragment of *FNDC5*, increased the release of glycerol and *ATGL* and *HSL* mRNA abundance in bovine and human adipose tissue explants, thus directly stimulating adipose tissue lipid mobilization.

Irisin was identified as a proteolytic cleavage product of FNDC5, a transmembrane protein present mainly in skeletal muscle (32, 45). *FNDC5* mRNA is most abundantly expressed in human skeletal muscle and in the muscle-rich pericardium and rectum. It is also highly expressed in the heart, the tongue, the intracranial artery, and the optic nerve (46). However, it has been reported that irisin is also produced by human and rodent adipocytes (47, 48). *FNDC5* gene is expressed in muscle at levels much higher than in adipose tissue in humans (49), mice (50) and cattle (51). However, in humans, it is the expression of *FNDC5* in adipose tissue and not skeletal muscle that correlates with concentrations of circulating irisin (47). In contrast, in our present study, we detected greater abundance of FNDC5 mRNA and protein in subcutaneous adipose tissue compared with skeletal muscle. This discrepancy is not easy to explain, but may be related to site of tissue sampling [semitendinosus muscle in our study vs. longissimus muscle in Komolka et al. (51)], sex of the individual [male cattle in (51)] or breed (dairy vs. beef cattle). In humans, a sexual dimorphism in irisin concentrations at rest has been described with a higher concentration in girls vs. boys (52) and in women vs. men (53). In rats, low-oxidative fiber-type muscle (soleus) secretes ~40% more FNDC5/irisin than fast-glycolytic fiber-type muscle (gastrocnemius) (48). Western blot experiments demonstrated the presence of the full-length transmembrane protein (~25 kDa) and not the cleaved form (~12.6 kDa), which is to be expected in tissue samples as the cleaved form would be released from the cell surface and presumably into the circulation. Our data are in a good agreement with those of Boström (32) and Roca-Rivada (48) who detected a predominant band of ~25 kDa especially when using an anti-irisin antibody (anti-Irisin Phoenix Pharmaceuticals amino acids 42–112). As reported by Roca-Rivada et al. (48), the full length FNDC5/irisin is also probably secreted. To our knowledge, the release of the 12.6 kDa form of irisin from muscles or other tissues has not been reported so far.

It is known that the secretion of several adipokines changes during the NEB of early lactation, including resistin that increases and adiponectin that decreases at the onset of lactation (13, 17), and the present data show that members of the FNDC family are also affected. In the present study, abundance of adipose *FNDC5* mRNA and plasma irisin/FNDC5 concentrations were increased during early lactation concomitant with increased plasma NEFA concentrations and adipose tissue mobilization. Plasma FNDC5 is correlated with *FNDC5* mRNA levels and *FNDC5* protein expression in adipose tissue during the peripartum period. Generally, mRNA expression is assumed to be predictive of protein expression level. However, the correlation between mRNA and protein expression has been described as moderately or weakly positive (correlation coefficients ranging from 0.2 to 0.6), especially with regard to secreted proteins (54, 55). Our results illustrate this fact well. The question is now to understand what is the origin of the increase in plasma FNDC5 particularly at 1 WPP. We can hypothesize that the increase in plasma FNDC5 concentration is due to secretion from skeletal muscle but unfortunately we did not perform muscle biopsy in our protocol to verify this hypothesis. In our study, adipose *FNDC5* mRNA abundance was higher in animals with a deeper negative energy balance 1 WPP. In human and rodent models, there is no clear association between nutrition and FNDC5 expression, as in some studies adipose *FNDC5* mRNA abundance is increased by exercise and in lean compared with obese subjects (56), whereas plasma irisin concentrations decrease after rapid weight loss in some studies (56) but not in others (57). The lactating dairy cow is an example of sudden changes in energy balance, and the increase in *FNDC5* mRNA and plasma irisin/FNDC5 concentrations observed here may be an adaptive response to this NEB. This is supported by the present data showing that irisin increased lipolysis in bovine adipose tissue explants suggesting that irisin could be involved in fat mobilization. A similar effect was observed in human adipocytes (58) and in 3T3-L1 adipocytes (59, 60), although other studies have reported no effect or a decrease in lipolysis (61, 62). Resistin also increases lipolysis (9), suggesting that a number of adipokines are involved in the mobilization of reserves to meet the energy demands of early lactation.

Three other FNDC family members were shown to be altered during NEB in the present study, FNDC3A, FNDC3B and *FNDC4*. All three were found to be widely expressed in bovine tissues, although abundance of FNDC4 mRNA in adipose tissue was very low suggesting that this FNDC member is a myokine rather than an adipokine. Although mRNA abundance of all three was increased in adipose tissue during NEB, there were no changes in plasma concentrations. In humans, *FNDC4* mRNA abundance in adipose tissue is higher in obese than in lean subjects whereas plasma FNDC4 concentrations are lower in obese patients (40); this, and the present data, suggests that adipose tissue is not a major contributor to plasma concentrations, and are consistent with the finding that the liver is the major source of circulating FNDC4 (63). The source of circulating FNDC3A and FNDC3B is unclear as these transmembrane proteins are not known to be cleaved and released as a secreted hormone; it is more likely that adipocyte (and other) cell death released membrane proteins into the circulation, and that this is not linked to adipose tissue mobilization during NEB.

In conclusion, the present data show that *FNDC5* plasma concentrations and FNDC5 gene expression in adipose tissue are increased 1 week after calving when the mobilization of reserves is high. Furthermore, FNDC5 is expressed in dairy cow muscle and subcutaneous adipose tissue and irisin may enhanced lipid mobilization in bovine adipose explants. Thus, we can hypothesize that increased irisin is an adaptive response to NEB and serves to meet the energy demands of lactation in cattle.

## Data availability statement

The raw data supporting the conclusions of this article will be made available by the authors, without undue reservation.

## Ethics statement

The studies involving human participants were reviewed and approved by Patient wrote consent and after local Ethical Committee agreement (CNIL no. 18254562). The patients/participants provided their written informed consent to participate in this study. The animal study was reviewed and approved by Comité d'Ethique en Expérimentation Animale Val de Loire, CEEA VdL Number 19, protocol reference number 2012-10-4.

## Author contributions

MD and CR: methodology, validation, formal analysis, investigation, visualization, and writing—original draft. AE: methodology and writing—review and editing. CP: methodology, formal analysis, visualization, writing—review and editing, and supervision. JD: methodology, validation, formal analysis, investigation, visualization, writing—review and editing, supervision, project administration, and funding acquisition. All authors contributed to the article and approved the submitted version.

## Funding

This work was financially supported by INRAE and RQR (Réseau Québécois en Reproduction). The research leading to these results has received funding from the European Union Seventh Framework Programs (FP7: 2007-2013) under the grant agreement no. 311776 and from the Fonds de Recherche du Québec - Nature et Technologies, administered through the Réseau Québécois en Reproduction.

## Conflict of interest

The authors declare that the research was conducted in the absence of any commercial or financial relationships that could be construed as a potential conflict of interest.

## Publisher's note

All claims expressed in this article are solely those of the authors and do not necessarily represent those of their affiliated organizations, or those of the publisher, the editors and the reviewers. Any product that may be evaluated in this article, or claim that may be made by its manufacturer, is not guaranteed or endorsed by the publisher.

## Abbreviations

NEB: negative energy balance
BHBA: β-hydroxybutyrate
NEFA: non-esterified fatty acid
FNDC: fibronectin type III domain-containing
WAP: week ante-partum
WPP: week post-partum
LNEB: low negative energy balance
HNEB: high negative energy balance
ATGL: adipose triglyceride lipase
HSL: hormone sensitive lipase
PPARγ: peroxisome proliferator activated receptor γ
UCP1: uncoupling protein 1
ITGB1: integrin β1
ITGAV: integrin αV
MAPK3/1: mitogen-activated protein kinase 3/1
Fad104: factor for adipocyte differentiation 104
ADGRF5: adhesion G-protein-coupled receptor F5
GPR116: G-protein-coupled receptor 116
EB: energy balance
DMI: dry matter intake
MY: milk yield
LBW: live body weight
EBW: empty body weight
BFT: back fat thickness.
